# Effects of Web-Based Social Connectedness on Older Adults’ Depressive Symptoms: A Two-Wave Cross-Lagged Panel Study

**DOI:** 10.2196/21275

**Published:** 2021-01-13

**Authors:** Juwon Hwang, Catalina L Toma, Junhan Chen, Dhavan V Shah, David Gustafson, Marie-Louise Mares

**Affiliations:** 1 School of Journalism and Mass Communication University of Wisconsin Madison, WI United States; 2 Department of Communication Arts University of Wisconsin-Madison Madison, WI United States; 3 Department of Communication University of Maryland College Park, MD United States; 4 Center for Health Enhancement Systems Studies University of Wisconsin Madison, WI United States

**Keywords:** depressive symptoms, older adults, web-based intervention, online social support, patient health questionnaire, longitudinal survey, mobile phone

## Abstract

**Background:**

Depressive symptoms are the most prevalent mental health concern among older adults (possibly heightened during the COVID-19 pandemic), which raises questions about how such symptoms can be lowered in this population. Existing research shows that *offline* social connectedness is a protective factor against depression in older adults; however, it is unknown whether *web-based* social connectedness can have similar effects.

**Objective:**

This study investigates whether social connectedness on a support website protects older adults against depressive symptoms over the course of a year, above and beyond the protective effect of offline social connectedness. The secondary aim is to determine whether older adults with increased depressive symptoms are more likely to engage in social connectedness on this website. Thus, we examine depressive symptoms as both an outcome and predictor of web-based social connectedness to fully understand the chain of causality among these variables. Finally, we compare web-based social connectedness with offline social connectedness in their ability to lower depressive symptoms among older adults.

**Methods:**

A total of 197 adults aged 65 years or older were given access to a social support website, where they were able to communicate with each other via a discussion forum for a year. Participants’ social connectedness on the web-based platform, conceptualized as message production and consumption, was measured using behavioral log data as the number of messages participants wrote and read, respectively, during the first 6 months (t_1_) and the following 6 months (t_2_) of the study. Participants self-reported their offline social connectedness as the number of people in their support networks, and they reported their depressive symptoms using the Patient Health Questionnaire-8 both at baseline (t_1_) and at 12-month follow-up (t_2_). To ascertain the flow of causality between these variables, we employed a cross-lagged panel design, in which all variables were measured at t_1_ and t_2_.

**Results:**

After controlling for the effect of offline support networks at t_1_, web-based message consumption at t_1_ decreased older adults’ depressive symptoms at t_2_ (*β*=−.11; *P*=.02), but web-based message production at t_1_ did not impact t_2_ depressive symptoms (*β*=.12; *P*=.34). Web-based message consumption had a larger effect (*β*=−.11; *P*=.02) than offline support networks (*β*=−.08; *P*=.03) in reducing older adults’ depressive symptoms over time. Higher baseline depressive symptoms did not predict increased web-based message consumption (*β*=.12; *P*=.36) or production (*β*=.02; *P*=.43) over time.

**Conclusions:**

The more messages older adults read on the web-based forum for the first 6 months of the study, the less depressed they felt at the 1-year follow-up, above and beyond the availability of offline support networks at baseline. This pinpoints the substantial potential of web-based communication to combat depressive symptoms in this vulnerable population.

**International Registered Report Identifier (IRRID):**

RR2-10.1186/s13063-015-0713-2

## Introduction

### Background

The number of people aged 65 years and older is expected to rise dramatically over the next few decades, accounting for 16.7% of the global population by 2050 [1] and 23.5% of the US population by 2060 [2]. The most prevalent mental health problem among older adults is depression [3], encompassing both major depression and significant depressive symptoms that are below the severity threshold of major depression [4]. Although events such as COVID-19 stay-at-home directives can play a significant role, the multiple changes that occur in late adulthood are largely responsible for depressive symptomology in older adults [5]. For instance, older adults are likely to experience poor health and physical constraints [6] and transition in roles [7], and some older adults experience financial difficulties [6] and social isolation due to retirement and death of friends and family [7]. Both major depression and depressive symptoms cause significant disruption in the daily lives of older adults. Depression is strongly associated with more chronic diseases [4,8], high functional disability and mortality [9], and low quality of life [10]. The prevalence of depressive symptoms among older adults and their harmful consequences underscore the need to understand factors that may guard against the development or continuation of depressive symptoms in this age group.

One such factor is social connectedness [11,12]. Research shows that higher-quality social ties and meaningful social interactions significantly reduce depression in older adults [13-16]. However, the existing literature focuses exclusively on *offline* social connectedness. With internet adoption skyrocketing among this demographic [17], an intriguing possibility is that *web-based* social connectedness has similar protective effects against older adults’ depressiveness. In particular, a robust literature shows that online support groups provide options for social connectedness that substantially enhance users’ psychological well-being (for a review, see the study by Wright [18]), although research on older adults’ participation in these groups is limited and has not yet tackled the issue of depressive symptoms. Therefore, we ask: Does social connectedness via online support groups protect older adults against depressive symptoms above and beyond the well-documented protection provided by social connectedness?

A long-standing difficulty in researching the relationship between social connectedness and depressive symptoms is elucidating the chain of causality. Does social connectedness affect depressive symptoms, or does depressive symptoms affect social connectedness, or both? Indeed, in the realm of *offline* social connectedness, studies show that social connectedness correlates with reduction in depressive symptoms [13,14,16,19], indicating that depressive symptoms can be conceptualized as an outcome of social connectedness. However, other studies show that depressive symptoms correlate with individuals’ tendency to withdraw from social situations [20-22], indicating that depressive symptoms can also be conceptualized as an antecedent of social connectedness. Few studies take an integrative approach that considers both causal paths [23,24]. Here, we collect longitudinal data with key variables measures at two time points, which enables us to simultaneously test both causal pathways. Therefore, we are able to make causal inferences about whether web-based social connectedness improves older adults’ depressive symptoms over time, and whether older adults are likely to reap the benefits of web-based social connectedness by gravitating toward web-based opportunities for social connection when they experience depressive symptoms.

We begin with a brief review of the benefits of *offline* social connectedness for mitigating depressive symptoms in older adults. Then, we discuss how *web-based* social connectedness may benefit older adults above and beyond the well-established benefits of offline connectedness.

### Offline Social Connectedness and Depressive Symptoms Among Older Adults

Considerable research has shown that offline social connectedness is associated with lower depressive symptoms in the older population [13-16]. Importantly, these benefits do not stem from the sheer number of social connections: individuals with large social networks are not immune to loneliness and depression [25,26]. Instead, it is only close, supportive relationships that protect against depressive symptoms [11-13], because they provide individuals with a sense of being cared for, loved, and valued [13,20,27]. Much of this research has relied on cross-sectional designs, which limit causal inference. Nonetheless, a couple of longitudinal studies provide indication of causality. The loss of a spouse [13] and limited in-person contact with family and friends [14] were found to increase depressive symptoms in older adults over time, suggesting that the presence of supportive companions acts as a buffer against depression in this population. In light of this evidence, we expect that the availability of offline support networks will lead to reductions in older adults’ depressive symptoms over time.

### The Added Benefit of Social Connectedness on Support Websites

Does social connectedness on support websites contribute to older adults’ well-being above and beyond offline connectedness? Support websites have become a popular and effective tool for linking individuals with others who share similar life difficulties, yet whom they may never have encountered in everyday life [28]. These websites are rooted in the same principles as those of traditional face-to-face support groups, namely that people’s mental and physical health can be bolstered when they perceive their situation as universal rather than unique, when they share information that can help others, and when they receive supportive messages from peers [29-34]. An additional reason for support websites’ effectiveness is that they are anonymous and asynchronous, which enables participants to be more forthcoming about difficult, embarrassing, or stigmatizing topics [18,32].

Although the benefits of participation in online social support groups are well documented for those who have health issues such as cancer [30-32], diabetes [35], and HIV or AIDS [36], no research to our knowledge has investigated older adults’ social connectedness in these groups in relation to their depressive symptoms. A small body of literature shows that retirement, family, and health ranked among the most popular topics discussed by older adults in web-based communities [37], but that older adults also expressed empathy for other members [38] and developed closely connected subnetworks based on emotional communication [39] in these interaction spaces. This suggests that support websites are conducive to the formation of social bonds between older adults and hold potential for reducing depressive symptoms in this population.

Connecting with peers on support websites can take 2 forms: (1) posting messages and responses to other users’ messages (ie, *message* *production*) and (2) reading other users’ postings, thus keeping abreast of others’ experiences and insights (ie, *message consumption*). To date, most studies have investigated these 2 forms of social connection separately, finding them both beneficial for participants’ well-being [30-32]. Writing messages was shown to alleviate depression, loneliness, pain, and stress among younger individuals [18,40-42]. These benefits accrued because writing in a supportive setting helped participants to reframe their problems in a positive manner and adopt positive coping strategies [43]. Consuming messages by others who were experiencing similar struggles helped participants reduce worry and distress [43,44] because it made them feel less isolated in their struggles [44] and exposed them to different perspectives on a given problem [30]. It bears noting that, contrary to these findings, lurking on social network sites (ie, monitoring others’ behaviors without directly communicating with them) has been associated with reductions in well-being [45]. This is likely the case because users of social network sites tend to post glamorized presentations of their lives, thus eliciting envy from viewers [46]. In contrast, users of online support groups tend to show their vulnerabilities and provide encouragement, useful information, and a sense of community to others [47]. Indeed, message consumption on online support groups appears to be helpful even in the absence of message production: participants who read many messages but seldom wrote their own (ie, lurkers [44,48]) experienced increased belonging to the group [48] and reduced isolation [44]. Building on this work, we hypothesize that older adults will also benefit from both web-based message production and consumption within an online support group. These 2 forms of engagement should facilitate the formation of supportive bonds, which in turn should reduce depressive symptoms in this population:

Hypothesis 1. Message production and consumption on a support website will lead to reductions in older adults’ depressive symptoms a year later, above and beyond reductions generated by the availability of offline support networks.

### The Effects of Web-Based Connectedness Versus Offline Connectedness on Depressive Symptoms

We expect that both web-based and offline social connectedness will serve as protective factors against depressive symptoms in older adults. This raises the question of the relative contribution of these 2 types of connectedness to the amelioration of depressive symptoms.

Research shows that feelings of social connectedness derived from web-based interaction are distinct from those derived from face-to-face interaction, in part because some users connect with different networks online than they do offline. Thus, it is possible to feel connected to web-based networks while disconnected from face-to-face networks and vice-versa [11,49]. In our case, it is unlikely that web-based and offline networks overlap: web-based connections developed on social support websites are likely to be new additions to older adults’ offline support networks, rather than duplicates. Nonetheless, both types of connectedness have been shown to be effective in boosting well-being [12,49-51]. As no research has compared the effects of web-based and offline connectedness on alleviating depressive symptoms in general or older adults’ depressive symptoms in particular, we pose the following research question:

Research question 1. Is web-based social connectedness or offline social connectedness more potent in reducing depressive symptoms among older adults after 1 year?

### Web-Based Connectedness, Depressive Symptoms, and Causal Inference

Finally, let us now consider the issue of chain of causality. As previously hypothesized, we expect psychological benefits to accrue to older adults who connect socially via online support groups. In addition, it is possible that older adults’ depressive symptoms affect the extent to which they engage in web-based social connection to begin with. In offline settings, individuals with depression have been shown to withdraw from social situations because of fatigue, lethargy, or diminished feelings of self-worth [20-22]. Depressive symptoms are also predictive of reduced offline connections because depressed individuals’ negative self-statements, complaints, and social inadequacy often alienate others [20-22].

To compensate for a lack of offline connectedness, individuals with depressive symptoms might turn to web-based communication, which is substantially less effortful (ie, it can be done without leaving the house) and easier to control because of web-based affordances such as editability and unlimited time to compose messages [52]. Indeed, the social compensation hypothesis [53] predicts that web-based communication is preferred by individuals who experience psychosocial problems that hinder their ability to engage in meaningful face-to-face interactions (eg, anxiety, loneliness, and depression), precisely because it is easy and convenient, and provides an enhanced sense of control over message production and consumption [53,54]. In support of this contention, research shows that participants experiencing more depression tended to increase their engagement with online support groups by posting messages [44] and seeking help from those who experienced similar health issues [29]. Therefore, we predict that older adults with more depressive symptoms are more likely to be engaged in reading and writing messages within an online social support group:

Hypothesis 2. Older adults experiencing more depressive symptoms will engage in greater message production and consumption within an online social support group over time.

## Methods

### Study Context

The data comes from a clinical trial of an online social support group intervention for older adults named Elder Tree [55]. The intervention consisted of providing participants access to a support website. This website was designed to enable older adults to provide and receive peer support through a web-based discussion forum, with the ultimate goal of helping them maintain their independence and health. The discussion forum enabled older adults to communicate with one another by starting and responding to threads or simply reading each other’s messages. Sample messages are provided in Multimedia Appendix 1.

Topics in the forum were not limited to any particular health concerns; rather, they ranged broadly from health to politics or religion. The forum was primarily text-based, although it also supported the uploading of photographs. It was asynchronous, meaning that participants could take all the time they wanted to compose and read messages. Posts could be pseudo-anonymous, meaning that participants were able to express themselves anonymously if they wished, but were also free to disclose their real names and personal photographs. Trained facilitators monitored the conversations to ensure that discussions were supportive and did not contain unchallenged inaccurate or harmful information; however, they did not play an active role in guiding the topics of conversation. Communication took place in a public section, where participants could post messages viewable to all members, as well as in a private section, where participants could post messages only viewable to specific individuals.

### Participants and Procedure

Participants were adults aged 65 years and above who had experienced one or more of the following in the last 12 months: a fall, feeling sad or depressed, home-health services, a stay in a skilled nursing facility, an emergency room visit, or admission to the hospital. As our focus was on older adults aging in place, participants were excluded if they were homeless; lived in a hospice center, assisted living facility, or a nursing home; or needed help getting in or out of a bed or a chair. Participants were recruited through senior centers, churches, other community groups, and Aging and Disability Resource Centers. Research coordinators assessed older adults who volunteered for our study; mailed eligible participants the baseline survey; and made home visits to explain the study and obtain consent, collect the baseline survey responses, and explain to participants the expectations of the study based on the group to which they were randomized. Other team members visited participants to give them computers, if needed, and train them on using the website. The training included tutorials on how to use the computer and the internet, as required, but focused on the use of the various features of the support website. Participants were recruited from November 2013 to May 2015, with the intervention ending in November 2016.

Our analyses were conducted on the sample of 197 participants (145/197, 73.6% women; age range: 65-100 years, mean 76.26, SD 7.38) who were given access to the support website. After 12 months, the sample was reduced to 159 due to attrition (80.7% retention rate). It must be noted that the data come from a clinical trial of a support website, where participants were randomly assigned to either access this website or not [55]. In this analysis, only participants who had access to the website were included.

### Study Design

This study was set up as a 2-wave cross-lagged panel design, with all variables of interest (ie, depressive symptoms, offline support network size, web-based message consumption, and web-based message production) measured at both time points. The cross-lagged paths allow us to assess causal links between variables over time. As this design allows controlling for variables measured at a previous time point, it rules out the alternative explanations that may occur in cross-sectional studies such as reverse causality and the influence of an auto-regressor [56]. Cross-lagged panel designs are considered the optimal way to understand causality among naturally occurring variables in field settings where experimental procedures are not feasible [57]. Finally, as each person is his or her own control and results can be interpreted as pertaining to the relationship between within-person changes, this design does not require the inclusion of other covariates [58] (see Multimedia Appendix 2 [59,60] for more information on cross-lagged panel designs).

### Measures

This study examined longitudinal data with 2 major components: (1) self-reported surveys that were administered at baseline and at the 12-month follow-up and (2) behavioral log data of website use during the study period.

### Survey Data

*Depressive symptoms* were assessed with 8 items of the Patient Health Questionnaire (PHQ) [61] on a 4-point scale (0=not at all, 3=nearly every day) at both time points. Participants rated the degree to which they experienced little interest or pleasure in doing things; feeling down, depressed, or hopeless; and trouble falling or staying asleep, or sleeping too much, for example. The ratings for each item were summed to produce a total score between 0 and 24 points (t1: α=.87; mean 4.42, SD 4.65; t2: α=.87; mean 3.91, SD 4.29). Higher scores indicate higher depressive symptoms. On the basis of the depressive symptom score at baseline, participants were classified into the following depressiveness groups: none to minimal (ie, 0-4 score; 125/197, 63.5% of the participants), mild (ie, 5-9 score; 47/197, 23.8% of the participants), moderate (ie, 10-14 score; 15/197, 7.6% of the participants), moderately severe (ie, 15-19 score; 7/197, 3.6% of the participants), and severe (ie, 20-24 score; 3/197, 1.5% of the participants). Dropout analyses indicated no significant differences in baseline depressive symptoms between the participants in the final sample and those who dropped out between t1 and t2.

*The offline support network* was assessed with 2 items at both time points. Participants indicated the number of people *you can count on to listen to you when you need to talk and count on you to listen to them when they need to talk*. These items were averaged to produce an overall score (t1: α=.89, mean 4.95, SD 4.80; t2: α=.70, mean 6.21, SD 8.32). Examination of kurtosis values indicated the presence of an outlier case, yielding the ability to skew overall results [62]. The outlier value was winsorized by transforming it with the largest value in observations, excluding the outlier [63,64]. Although outliers are often removed from data, winsorization is recommended for analyses with a small sample size [63].

### Behavioral Log Data

Participants’ use of the website was captured automatically at an individual keystroke level, as participants used the system. The browser produced a log file with each participant’s username, date, and URL of every web page requested from the web server. This enabled us to track the number of messages each participant wrote and/or read in the discussion forum. Recall that in the survey data, t_1_ is defined as the survey participants filled out at baseline, whereas t_2_ is defined as the survey they filled out at the 12-month follow-up. With the behavioral log data, t_1_ is defined as participants’ web-based engagement during the first 6 months (baseline to month 6), and t_2_ is defined as their engagement during the last 6 months (from month 6 to month 12).

*Web-based message consumption* was operationalized as the number of messages participants viewed in the peer-to-peer discussion forums during the first 6 months of participation (t1: mean 1063.87, SD 3478.41) and the following 6 months (t2: mean 700.54, SD 2423.15).

*Web-based message production* was operationalized as the number of messages participants created in the discussion forum for the first 6 months (t1: mean 23.80, SD 78.45) and the following 6 months (t2: mean 28.11, SD 106.37).

### Data Analysis

**Figure 1:**
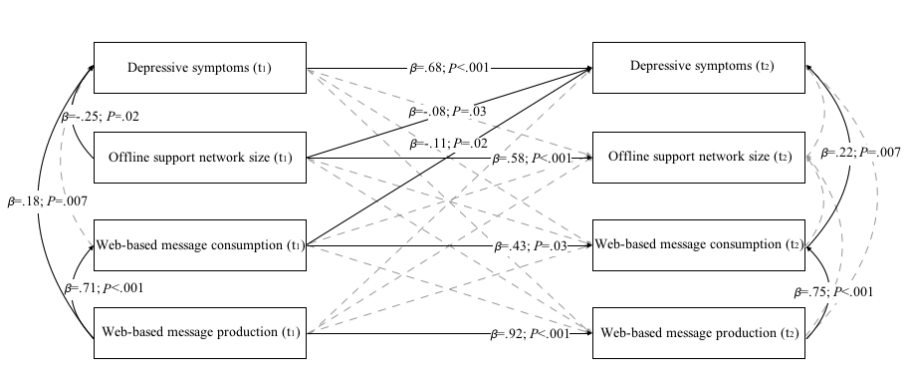
Cross-lagged path model of observed long-term effects on depressive symptoms, offline support network size, web-based message consumption, and web-based message production over 1 year. Nonsignificant paths are represented as dashed lines with muted colors without their coefficients.

To test the hypotheses, depressive symptoms, offline support network, web-based message consumption, and web-based message production measured at t_1_ were allowed to simultaneously predict these same constructs at t_2_ in a cross-lagged panel design. The 4 exogenous variables at t_1_ and the 4 endogenous variables at t_2_ were specified to correlate. Most relevant for evaluating our hypotheses are the cross-lagged paths between the constructs assessed at both t_1_ and t_2_ (Figure 1). Models were tested using a full information maximum likelihood estimation, in which missing data were imputed [64]. Given that web-based message consumption and production data were highly skewed, we used maximum likelihood estimation with robust standard errors to address the nonnormality problem.

Fully cross-lagged models require the saturated estimation of all possible path combinations, but this would produce just-identified models containing 0 degrees of freedom. This makes it impossible to obtain model fit indices [65]. To obtain optimal model fit indices and achieve parsimony, it is recommended to undertake post hoc modification, whereby nonsignificant and theoretically uninformative paths are removed [56]. Thus, we removed 2 paths showing insignificant relationships between exogenous variables (ie, web-based message consumption at t_1_ and offline support network at t_1_ and web-based message production at t_1_ and offline support network at t_1_).

The resulting model showed an excellent fit to the data (*χ*^2^_27_=273.9 root mean square error of approximation=0.04; standardized root mean square residual=0.01; comparative fit index=0.99; and Tucker-Lewis Index=0.96). The explained variance (*R*^2^) for depressive symptoms, offline support network, web-based message consumption, and web-based message production at t_2_ were 57.6%, 34.1%, 68.8%, and 81.3%, respectively.

## Results

### Descriptive Statistics

Descriptive characteristics of participants at baseline are presented in Table 1. Means, SDs, and person correlation coefficients between all variables that were subsequently included in the cross-lagged models are reported in Table 2. Participants’ reports of depressive symptoms, offline support networks, web-based message production, and web-based message consumption did not change over time.

**Table 1:** Descriptive characteristics of participants at baseline (N=197).

Characteristics		Values
Age, years, mean (SD)		76.26 (7.38)
**Gender, n (%)**		
	Male	52 (26.4)
	Female	145 (73.6)
**Race or ethnicity, n (%)^a^**		
	White	176 (89.3)
	Black	19 (9.6)
	Other	8 (4.1)
**Education, n (%)**		
	Less than high school	0 (0.0)
	Some high school or diploma	74 (37.6)
	Some college or post-high school	68 (34.5)
	4-year degree or above	55 (27.9)
**Living arrangement, n (%)^a^**		
	Living alone	121 (61.4)
	Spouse or partner	61 (31.0)
	Son or daughter	15 (7.6)
	Other family or friends	3 (1.5)
	Paid caregiver	1 (0.5)
	No response	1 (0.5)
**Comfort with technology, mean (SD)^b^**		
	Smartphone or tablet	1.4 (1.8)
	Desktop computer	3.2 (1.8)
	Email	2.8 (2.1)
	Facebook	1.7 (2.0)

^a^Group totals may exceed 100% because participants could report more than one race or ethnicity and living arrangement.

^b^Comfort with technology was measured with a 6-point scale (0=*never used* to 5=*very comfortable*).

**Table 2:** Means, SDs, and Pearson correlation coefficients for key variables at both wavesa.

Variables	Mean (SD)	1. Depressive symptoms (t_1_)	2. Depressive symptoms (t_2_)	3. Offline support network size (t_1_)	4. Offline support network size (t_2_)	5. Web-based message consumption (t_1_)	6. Web-based message consumption (t_2_)	7. Web-based message production (t_1_)	8. Web-based message production (t_2_)
1. Depressive symptoms (t_1_)	4.42 (4.65)	1	—^b^	—	—	—	—	—	—
2. Depressive symptoms (t_2_)	3.91 (4.29)	.76^c^	1	—	—	—	—	—	—
3. Offline support network size (t_1_)	4.95 (4.80)	−.25^c^	−.28^c^	1	—	—	—	—	—
4. Offline support network size (t_2_)	6.21 (8.32)	−.17^d^	−.22^e^	.61^c^	1	—	—	—	—
5. Web-based message consumption (t_1_)	1063.87 (3478.41)	.03	.04	−.02	−.00	1	—	—	—
6. Web-based message consumption (t_2_)	700.54 (2423.15)	.18^e^	.23^e^	−.03	−.00	.69^c^	1	—	—
7. Web-based message production (t_1_)	23.80 (78.45)	.09	.10	.03	−.01	.71^c^	.70^c^	1	—
8. Web-based message production (t_2_)	28.11 (106.37)	.10	.10	.04	−.01	.62^c^	.76^c^	.90^c^	1

^a^Although all variables were denoted as t_1_ and t_2_, the specific time points varied—for depressive symptoms and offline support network size, t_1_ and t_2_ indicate baseline and follow-up 12 months later, respectively. For web-based message consumption and production, t_1_ and t_2_ indicate the first 6 months (baseline to month 6) and the last 6 months of the study (from month 6 to month 12), respectively.

^b^The correlation coefficient was not shown as it was shown in the asymmetrically diagonal position of the table.

^c^Correlations significant at the 0.001 level.

^d^Correlations significant at the 0.05 level.

^e^Correlations significant at the 0.01 level.

### Hypothesis Testing

First, the cross-lagged relationship between older adults’ offline social connectedness at baseline (t_1_) and their depressive symptoms a year later (t_2_) was examined to replicate previous findings. Results confirmed that larger offline support networks at baseline significantly reduced older adults’ depressive symptoms a year later (*β*=−.08; *P*=.03).

A cross-sectional effect also emerged at baseline, with offline support networks negatively associated with depressive symptoms (*β*=−.25; *P*=.02), but no such correlation emerged a year later (*β*=−.06; *P*=.22). Substantial autocorrelations were found: depressive symptoms at baseline were significantly related to depressive symptoms a year later (*β*=.68; *P*<.001), and offline support network size at baseline was significantly associated with offline support network size a year later (*β*=.58; *P*<.001).

To test hypothesis 1, the cross-lagged relationship between older adults’ web-based message consumption and production for the first 6 months (t_1_) and their depressive symptoms a year after the study began (t_2_) was examined. A statistically significant negative lagged effect of web-based message consumption on depressive symptoms emerged (*β*=−.11; *P*=.02), but web-based message production had no lagged effect on depressive symptoms (*β*=.12; *P*=.34; Figure 1). This means that the more messages older adults read on the web-based forum during the first 6 months, the less depressed they felt a year after the study began, above and beyond the effect of offline support networks at baseline. However, the number of messages they wrote on the web-based forum during the first 6 months (t_1_) did not affect their depressive symptoms at the 1-year check marker (t_2_). Hypothesis 1 was partially supported.

Some cross-sectional effects between web-based connectedness and depressive symptoms emerged: greater depressive symptoms at baseline were associated with increased message production during the first 6 months of the study (*β*=.18; *P*=.007), but no such correlation emerged during the next 6 months (*β*=−.18; *P*=.14). Although there was no relationship between reading messages on the discussion board during the first 6 months and depressive symptoms at baseline (*β*=−.10; *P*=.11), participants who read more messages during the next 6 months experienced more depressive symptoms a year after the study began (*β*=.22; *P*=.007).

As expected, substantial autocorrelations were also found: web-based message consumption during the first 6 months of the study was significantly related to consumption during the next 6 months (*β*=.43; *P*=.03), and web-based message production during the first 6 months was significantly associated with production during the next 6 months (*β*=.92; *P*<.001). Web-based message consumption was correlated with production at both initial assessment (*β*=.71; *P*<.001) and 1 year later (*β*=.75; *P*<.001).

To address research question 1, the standardized coefficients for the significant lagged paths (ie, the effects of web-based message consumption during the first 6 months of the study [t_1_] on depressive symptoms a year after the study began [t_2_] and the effects of offline network size at baseline [t_1_] on depressive symptoms a year later [t_2_]) were compared [66]. Web-based message consumption had a considerably larger effect (*β*=−.11; *P*=.02) in reducing depressive symptoms 1 year after participation in the support website, compared with offline support network size (*β*=−.08; *P*=.03; Figure 1). To confirm that these 2 effects were in fact statistically different, the joint hypothesis test (*F* test) was performed. The results indicated that the difference between the 2 effects was significant (*F*_1,141_=55.242; *P*<.001).

Hypothesis 2 dealt with the issue of chain of causality, specifically whether depressive symptoms predict (in addition to being affected by) web-based connectedness. The results show no lagged effect of depressive symptoms on either web-based message consumption (*β*=.12; *P*=.36) or production (*β*=.02; *P*=.43), meaning that depressive symptoms at baseline did not play a role in older adults’ web-based connectedness during the next 6 months. Hypothesis 2 was not supported.

The data also allowed us to make inferences about the nature of the relationship between depressive symptoms and *offline* connectedness. The results show no cross-lagged effect of depressive symptoms on offline support networks (*β*=−.04; *P*=.43), meaning that baseline depressive symptoms did not lead older adults to lose their offline support networks over time.

## Discussion

### Principal Findings

The goal of this study was to understand the protective value of web-based social connectedness (ie, the extent to which older adults engaged with each other on a support website) against older adults’ depressiveness. We captured behavioral trace data (ie, the number of messages participants wrote and read on the support website) and participants’ self-reported depressiveness over the course of a year. The findings can be summarized as follows. Replicating prior research, the availability of offline support networks decreased older adults’ depressive symptoms a year later. Most importantly, aspects of web-based connectedness *further* reduced older adults’ depressive symptoms over time. Specifically, web-based message consumption, or the extent to which older adults read peers’ messages on the support website, reduced their depressive symptoms above and beyond the reduction generated by offline social connectedness. In fact, web-based message consumption had a larger effect than the availability of offline support networks in reducing older adults’ depressiveness. However, contrary to predictions, web-based message production, or the extent to which older adults wrote messages on the online discussion forum, did not affect their depressive symptoms a year later. Finally, initial depressive symptoms did not affect web-based message consumption or production, meaning that individuals who were more depressed initially did not seek out opportunities for social connection on the support website.

### Theoretical Implications

These findings contribute to the literature on several fronts. First, we advance research on older adults’ social connectedness and depressive symptoms, which, to date, has focused on traditional connections that originate in face-to-face settings (eg, family members and friends from the community). By shifting the lens toward *web-based* opportunities for connection, we found that low-effort participation on a support website, for instance simply reading messages generated by others experiencing similar life circumstances, afforded older adults significant protection against depressive symptoms. Our data do not address the mechanism behind this effect. However, likely candidates are suggested by research on the benefits of online social support. The key feature of support websites is that they operate via *homophily*, meaning that they connect individuals experiencing similar predicaments [18,34,47]. Although traditional face-to-face support networks, such as family members, typically wish to help, they often fail because they do not have lived experiences that would enable them to provide support seekers with a sense of being understood and validated in their struggles. For instance, research shows that younger individuals have a hard time understanding the challenges associated with aging [66]. Conversely, homophily-based networks by definition revolve around shared experiences. Simply witnessing others’ struggles and noting parallels with their own likely make older adults feel less alone [44] and part of a meaningful community [48]. In turn, feelings of inclusion and validation may reduce older adults’ depressive symptoms. This explanation is bolstered by the finding that web-based message consumption had even stronger effects in terms of reducing older adults’ depressiveness than the availability of offline support networks. Research finds that homophily increases mutual empathy and commitment to the group [34], explaining why older adults might feel a powerful sense of belonging with and validation from the virtual strangers who share similar experiences on the web. In addition, these virtual strangers became, over time, a daily presence in participants’ lives, with data showing that the majority of participants logged into the site on a daily basis. Even in the absence of actual interaction, this daily surveillance likely produced strong feelings of rapport. By contrast, older adults report significantly less frequent interactions with the members of their offline support networks, such as family and offline friends [67].

A related explanation for the benefits of web-based message consumption is that older adults in a support website could accrue informational support by taking the role of a passive user [47]. Informational support refers to advice, insights, tips, and other information that can help individuals manage difficult situations [18,68]. For example, older adults may learn about helpful medications, fitness activities, or community resources that can assist them. In turn, this informational support may help older adults adopt positive coping strategies, which could ultimately ameliorate their depressive symptoms.

Contrary to expectations, message production, or the extent to which older adults wrote messages on the online forum, did not buffer depressive symptoms. It is possible that older adults did not benefit from message production simply because they did not write much, preferring instead the less effortful activity of message consumption. Of the 197 participants, approximately 59 (29.9%) did not write any messages during the first 6 months of the study. The vast majority of messages (3774/4186, 90%) during this study period were written by only 10% (20/197) of the participants. It is likely that participants who wrote messages did, in fact, benefit from this activity. However, the small sample size of message writers in our dataset prevents us from statistically testing this possibility. Another possibility is that the therapeutic effect of writing does not last in the long run. Despite studies showing that writing helps immediately and as long as 2 weeks after writing [41,42], these benefits might dissipate over the course of a year.

Finally, our cross-lagged design was able to ask the reciprocal question of whether web-based social support was used as a resource by individuals who experienced greater depressive symptoms. The central tenet of the social compensation hypothesis [53] is that individuals use web-based communication to compensate for difficulties or missed opportunities in face-to-face settings. Extant research suggests that those manifesting depressive symptomology have a tendency to withdraw from offline interactions, either because they find them depleting or because they are shunned by face-to-face networks [20-22]; thus, web-based communication might serve as a resource for compensating for this social isolation. Neither of these predictions was borne out by these data. Depressive symptoms did not cause older adults to lose their offline support networks, nor did depressive symptoms increase the extent to which they engaged in web-based communication on the support website. Therefore, our finding that older adults with depressive symptoms did not gravitate toward web-based connectedness to satisfy social needs could be due to the fact that they did not have a shortfall to compensate for. In summary, this pattern of results suggests that depressive symptoms in older adults are best positioned as an outcome, rather than an antecedent, of web-based connectedness.

### Practical Implications for Online Social Support Group Design and Use

Online support groups demonstrated effectiveness in reducing older adults’ depressive symptoms in this study, indicating that this population should be encouraged to use them. Relatedly, health care systems might consider incorporating online support groups into programs that support the well-being of older adults, particularly those who are not able to attend face-to-face sessions. When it comes to the design of these support websites, our pattern of results, where message consumption (but not production) reduced older adults’ depressiveness over time, suggest that the presence of lurkers should be encouraged. Although active posting on these websites is certainly necessary, the lack of posting might not be as big of a problem as people think. Even lurking can have psychological benefits for older adults dealing with depressive symptoms.

Providing coaching to older adults on how to write effective web-based messages may encourage participants’ involvement in message production, from which they may obtain therapeutic benefits [41]. As suggested above, the nonsignificant effect of message production on depressive symptoms could be a consequence of most participants not writing messages. Coaching older adults may increase the number of messages they post in online support groups, allowing participants to be exposed to more of these beneficial messages.

These findings are even more important because of the COVID-19 virus. If older adults are isolated from their children and grandchildren for extended periods, this may have a negative impact on them. It is comforting to find that physical presence is not necessarily the only means by which support can be offered. The longer we have to maintain some forms of social distancing, the more important web-based vehicles for support are likely to become.

### Limitations and Future Directions

Several limitations should be addressed by future research. We operationalized social connectedness as the *quantity* of web-based (ie, number of messages read and posted) and offline (ie, number of individuals in support networks) engagement. Future research should complement our findings by investigating the content of web-based messages being read and written as well as the type of interactions older adults have with their offline networks. This will provide evidence of the mechanism through which social connectedness ameliorates depressive symptoms in older adults.

In this study, we used the label *offline* to denote relationships that were likely initiated offline (eg, with family members, work friends, neighbors, or community members), not relationships that are necessarily managed offline. It is possible that older adults’ communication with these networks occurs via mediated means, such as the telephone. Future research on how web-based connectedness can mitigate depressiveness among older adults should investigate mediated interactions with family members and friends made offline.

As participants were recruited on a rolling basis, they reported their depressive symptoms at different times. Given that there is evidence for seasonal fluctuations in mood or rates of depression [69], seasonality of depression may act as a potential confounder.

Finally, most participants in our sample were not clinically depressed. Only a fraction of our sample scored higher than the cutoff for clinical depression on the PHQ-8 questionnaire. However, the PHQ also captures individuals’ tendency to experience depressive symptoms that fall below the severity threshold for clinical depression, which was the case for our participants. Hence, we used the terminology *depressive symptoms* rather than *depression* throughout this manuscript. Future work should identify older adults with clinical depression to ascertain if the present findings hold for this more vulnerable group.

### Conclusions

Our research provides new insights into the protective power of web-based social connectedness against depressive symptoms among older adults by showing that reading others’ messages on a support website led to an amelioration of older adults’ depressive symptoms after 1 year of participation. This indicates that support websites might serve as a useful venue where older adults build an alternative form of social connection, which might be tremendously helpful for their psychological well-being.

## Appendix

Multimedia Appendix 1 Supplementary sample messages.

Multimedia Appendix 2 Supplementary descriptions about cross-lagged models.

## Abbreviations

PHQ: Patient Health Questionnaire
