# Supplementary material for: Effects of Web-Based Social Connectedness on Older Adults’ Depressive Symptoms: A Two-Wave Cross-Lagged Panel Study
Source: J Med Internet Res. 2021 Jan 13;23(1):e21275. doi: 10.2196/21275 (PMC7840281; doi:10.2196/21275)
Supplement: Multimedia Appendix 1 [file jmir_v23i1e21275_app1.docx]

Multimedia Appendix 1

Supplementary sample messages

Sample message 1: “Since my wifes illness, I'm the new cook on the block. I haven't learned any new quick meals, Just the old stand byes, Frozen pizza, hot dogs and beans, micro wave meals, Macacorni and cheese, and my favorite meals on wheels. I guess it will take me some time to really make a good home cooked meal. I am and will wait for any good suggestions.”

Sample message 2: “I see that we are building our group and I thought it might be good to introduce myself. My chat name is [ANONYMIZED] and I'm a caregiver for my father of 91 years of age. I have been caregiving for almost 2 years now for him in our home. My husband is very supportive as we believe it is the right thing to do. However, the right choice is not always the easiest, but this is truly a journey of various stages that I must learn as I go along the path to be a caregiver of a parent. I know it is not easy for my father being the recipient of my family's care either, but it brings him comfort knowing he is in a loving home that hopes to continue to care for him as long as we can. I must admit though balance is necessary. It's a bit tricky sometimes creating those boundaries to insure that I'm not living my life through his needs. So, education, patience, understanding and prayer are my resources. Hopefully my two grown sons are also learning something from this as our choice is not exactly one of this culture. I truly hope this resource of communication can assist us to help one another just through sharing our stories. Thanks for listening (reading)!”

Sample message 3: “How many of you readers are acquainted with Book Page? If you don't know about it, BookPage is a magazine (made of newsprint) that reviews books as they are published. It's a wonderful way to know what is new in the world of books. The Friends of the Waukesha Public Library provide many free copies at the self-check area. I always pick up one for myself and one for my daughter. The May issue is 31 pages, so you see there are lots of book reviews each month. It is categorized by fiction, non-fiction and children's. Those categories are further sorted into romance, mystery, cooking, science, etc. It's not just black and white, but colored, too, so you can visualize the book before you look for it on the shelves or in your book store. If you purchase a subscription, it will cost you $30 per year. They list a website of www.bookpage.com. I'm very happy that our Friends purchase so may copies for us in Waukesha.”
