# Supplementary material for: Effects of Web-Based Social Connectedness on Older Adults’ Depressive Symptoms: A Two-Wave Cross-Lagged Panel Study
Source: J Med Internet Res. 2021 Jan 13;23(1):e21275. doi: 10.2196/21275 (PMC7840281; doi:10.2196/21275)
Supplement: Multimedia Appendix 2 [file jmir_v23i1e21275_app2.docx]

Multimedia Appendix 2

Supplementary descriptions about cross-lagged models.

Cross-lagged models allow for the investigation of three types of relations [56,57]: (1) the synchronous correlations between two different constructs assessed at the same time point (e.g., depressive symptoms at t_1_ and offline support network size at t_1_), (2) auto-regression or temporal stability, which is defined as whether a variable predicts its subsequent measurement (e.g., depressive symptoms at t_1_ to depressive symptoms at t_2_), and (3) the cross-lagged paths of two conceptually distinct constructs assessed at different time points (e.g., offline support network size at t_1_ to depressive symptoms at t_2_).
